# Experimental infection of *Aphanomyces invadans* and susceptibility in seven species of tropical fish

**DOI:** 10.14202/vetworld.2015.1038-1044

**Published:** 2015-09-08

**Authors:** Seyedeh F. Afzali, Hassan Hj. Mohd Daud, Issa Sharifpour, Mohammad Afsharnasab, Shiv Shankar

**Affiliations:** 1 Department of Veterinary Clinical Studies, Faculty of Veterinary Medicine, Universiti Putra Malaysia, 43400 UPM, Serdang, Selangor, Malaysia; 2 Iranian Fisheries Research Organization, Tehran, Iran; 3 Department of Food Engineering, Mokpo National University, Korea

**Keywords:** *Aphanomyces invadans*, epizootic ulcerative syndrome, freshwater fish, histopathology

## Abstract

**Aim:**

Epizootic ulcerative syndrome (EUS) causes by aquatic oomycete fungus, *Aphanomyces invadans* is a dangerous fish disease of a wide range of fresh and brackish water, wild and farmed fish throughout the world. The objective of the present study was to determine the susceptibility of a number of tropical fish species to the EUS and compare the severity of infection between experimental groups.

**Materials and Methods:**

Snakehead, *Channa striata* (Bloch, 1793); snakeskin gourami, *Trichopodus pectoralis* (Regan, 1910); koi carp, *Cyprinus carpio* (Linnaeus, 1758); broadhead catfish, *Clarias macrocephalus* (Günther, 1864); goldfish, *Carassius auratus* (Linnaeus, 1758); climbing perch, *Anabas testudineus* (Bloch, 1792); and Nile tilapia, *Oreochromis niloticus* (Linnaeus, 1758) were challenged by intramuscular injection using zoospores of *Aphanomyces invadans* (NJM9701). The infected fish skins and muscles were examined for EUS histopathological characteristics, and the results on the severity of lesions and mortality were analyzed using SPSS program.

**Results:**

All zoospore-injected fish were shown to be susceptible to the EUS infection except Nile tilapia. Although, the general histopathological pattern was similar in the zoospore-injected group, but there were some variation in granulomatous reaction, that is the presence or absence of giant cells, and time of mortality were detected. The result of statistical analysis showed that there was a significant difference between species, (*c*^2^=145.11 and p<0.01).

**Conclusion:**

Gourami, koi carp, and catfish were demonstrated to be highly susceptible while goldfish and climbing perch were found to be moderately susceptible to the EUS infection. These findings suggested that the cellular response of fish to mycotic infection and granulomatous reaction varied in different fish species, which could not be an indicator of susceptibility or resistant to the EUS itself, although it was shown that the granulation rate and the level of maturity or solidification (consolidation of granulomas) were higher in resistant fish.

## Introduction

Epizootic ulcerative syndrome (EUS) is a clinical fish condition that arguably had the most serious socio-economic impact affecting the fresh and brackish water fish over the past 40 years and has been listed by the Aquatic Animal Code since 1995 [1]. The disease propagates through the countries of the Asia-Pacific region with dire consequences for fish resources and the livelihood of fishermen [2]. EUS is a disease that manifests in acute skin and muscle ulceration with the consequence of significant mortality in freshwater fish. The aquatic oomycete fungus, *Aphanomyces invadans*, has been identified as the causative agent of EUS [3,4] which releases a proteolytic enzyme facilitating penetration of fish tissue and precipitating shallow to deep ulcers, leading to a high mortality in fish population [5].

Until to date, no effective prophylactic measures and/or vaccines are available against EUS. If scientific development cannot solve this microbiological problem, it is likely to give significant negative impact on the income of fish farmers who rely on fish of wild origin for their livelihood. The potential effects of an introduction of the pathogen through international trade of tropical fish are latently hazardous.

In the current study, snakehead and Nile tilapia were used because of their importance as EUS-susceptible and resistant fish, respectively for the comparison of infection. The other freshwater fish species were selected for experimental infection due to their significant market demand in the Asian aquarium trade and food industry in order to determine their vulnerability to the causative agent of EUS and to compare the severity of infection in each species.

## Materials and Methods

### Ethical approval

The present study was approved by Institutional Animal Ethics Committee of the Universiti Putra Malaysia.

### Experimental challenges

For oomycete fungi culture and sporulation, hemp seeds were employed as bait. *Aphanomyces invadans* isolate NJM9701 (courtesy of Dr. Birgit Oidtmann) mycelia were encouraged to grow on sterile hemp seeds planted on the glucose-peptone (GP) agar. Subsequently, 6 mm growing hyphae on the hemp seeds were transferred into GP-yeast broth (GPY) with penicillin-streptomycin (10 ml/l) and incubated at 25°C for 5 days, then washed three times in autoclaved pond water (APW). The mycelium mats from the tip of colonies together with seeds were loaded into 1.5 ml microtubes containing APW for 24 h [6]. Fish utilized for challenge experimentation were snakehead, *Channa striata* (80±20 g); snakeskin gourami, *Trichopodus pectoralis* (12.5±2 g); koi, *Cyprinus carpio carpio* (25.5±4.5g); goldfish, *Carassius auratus* (16.5±1 g); broadhead catfish, *Clarias macrocephalus* (30±3.5g); climbing perch, *Anabas testudineus* (30±2 g); and Nile tilapia, *Oreochromis niloticus* (25±0.5 g). All fish were clinically healthy and kept in aquaria using de-chlorinated water equipped with air stones and aquarium heaters maintained at 20°C. Two replicate challenge groups and one control was set-up for each fish species utilizing 22 fish per tank. Fish were anesthetized using MS-222 (tricaine methanesulfonate) at 150 ppm prior to injection with 0.2 ml of a 10,000 spore/ml suspension (i.e. 2000 spores/fish) intramuscularly into the left side of the body below the dorsal fin. The control fish were inoculated with an equal volume of APW, and all fish were monitored daily for characteristic EUS clinical signs during the 35-days trial. Two fish from each tank were sampled at day 1, 2, 4, 6, 8, 10, 12, 14, 21, 28, 35 post-injection (p.i.) but dead and/or severely affected fish were collected as appropriate. Skin and muscle tissues (4-5 mm cubes) which excised from lesion areas were fixed in 10% phosphate-buffered formalin solution, processed through an automatic tissue processor and embedded in paraffin. The paraffin blocks were then sectioned at 4-5 µm thickness with a rotary microtome and mounted on clean glass slides. The slides were then stained with routine hematoxylin and eosin and periodic acid schiff (PAS) [7].

### Statistical analysis

**Figure-1:**
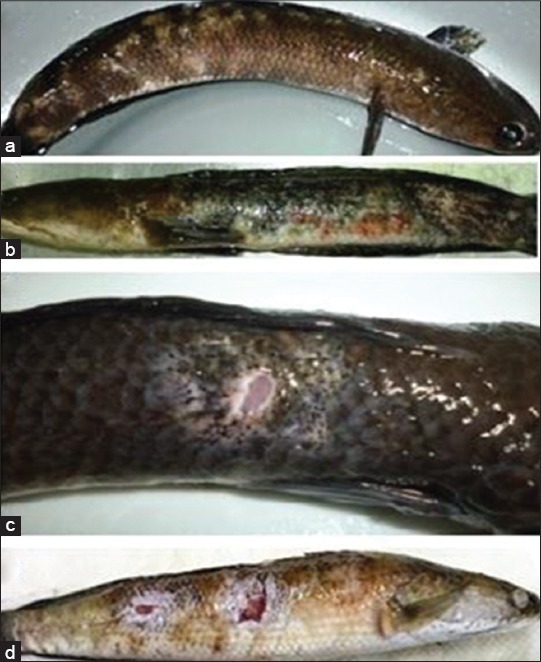
Visual description of skin lesion scoring system of examined fish is shown using epizootic ulcerative syndrome - affected snakehead lesions. (a) Score 1: Skin blanching lost of scale and epithelial cells. (b) Score 2: Red spot and marked swelling. (c) Score 3: Ulcerative lesion. (d) Score 4: Deep ulcers involving underlying muscles.

The severity of cutaneous lesions typical of EUS infection in fish was scored relative EUS lesions in snakehead, being a known an EUS-susceptible species (Figure-1). Data on the severity of lesions and mortality were analyzed using SPSS program. To compare between the experimental and control groups, a Chi-square test was applied, and the severity among seven fish species was compared using Kruskal–Wallis test.

## Results

### Experimental challenges

#### Gross observations

**Figure-2:**
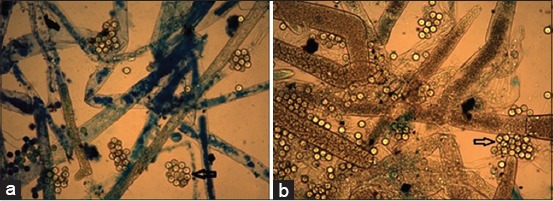
Smear preparation of epizootic ulcerative syndrome oomycete fungus “*Aphanomyces invadans*” re-isolated from artificially infected snakehead (a) and Snakeskin gourami (b). Typical *Aphanomyces invadans* non-septate hyphae are shown with cluster of encysted primary zoospores (arrows) (×100).

**Figure-3:**
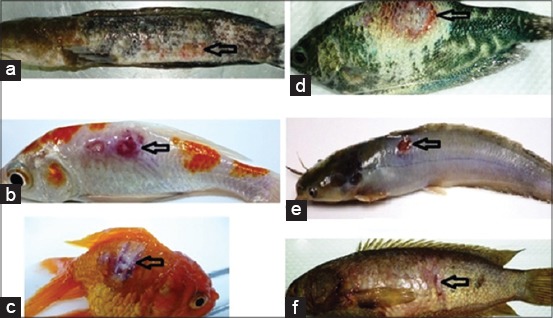
Epizootic ulcerative syndrome skin lesions (arrows) of fish injected artificially with *Aphanomyces invadans* spores: (a) Snakehead, (b) Snakeskin gourami, (c) Koi carp, (d) Broadhead catfish, (e) Goldfish, (f) Climbing perch.

Fish in the control group (APW-injected) did not show any behavioral or clinical signs of disease throughout the experiment. The positive identification of *A. invadans* aseptate hyphae (Figure-2) from the zoospore-injected tissues was carried out by the wet mount preparation method. EUS associated lesions were observed in all fishes inoculated with *A. invadans*, except in Nile tilapia (Figure-3). From 24 h after inoculation, the main clinical observations in zoospore-injected fish included localized swelling on the body and reddening. Scale loss and red ulceration underlying musculature were observed from day 4 of p.i. The red ulceration further developed into deep ulceration that was overlaid with fungal colonies from day 6 of p.i. Swimming behaviors were also affected, and most fish were observed swimming with a teethering movement, including unnatural backward and forward movement from day 8 of p.i. In some fish, the body color was affected, and it was observed that these fish showed pallor at the site of injection on day 10 of p.i. All moribund fish showed weakness with loss of appetite and remained at the bottom of the tank before death. In contrast to the other species which were used in this study, no visible inflammatory response was found in Nile tilapia, which was used as EUS-resistant fish during the course of the study.

### Histopathology

In both control and treatment groups, few degenerative changes due to the needle penetration were observed in all samples in the early stages of the disease, however, it was continued with inflammatory cell infiltration, especially macrophage response and severe invasive myonecrosis in *A. invadans* infected fish.

**Figure-4:**
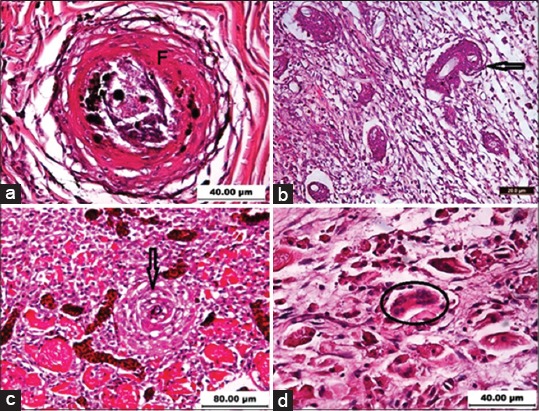
Histopathological characteristic of epizootic ulcerative syndrome-affected fish intramuscularly injected with *Aphanomyces invadans* NJM9701 zoospores. (a) Mature granuloma with necrotic center surrounded by fibroblast layers (F) in Snakehead (12 pi). Note the deposition of melanin pigments, H & E, 400×. (b) Formation of granulomata (arrow) characterized by thick fibroblast layers in Snakeskin gourami (10 pi), H & E, 200×. (c) A granuloma (arrow) surrounded by epithelioid cells in Koi carp (10 pi), H & E, 200×. (d) Foreign body type giant cells (circle) with surrounding connective tissues in broadhead catfish (14 dpi), H & E, 400×.

**Figure-5:**
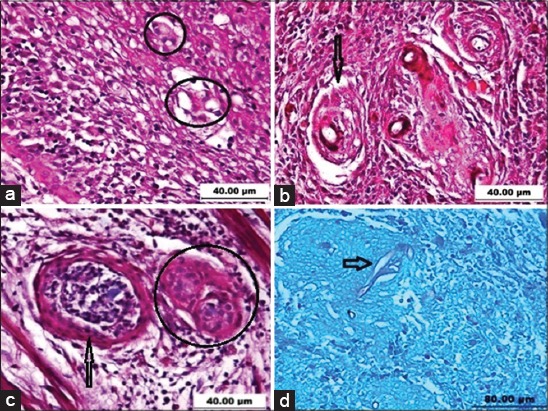
Histopathological characteristic of epizootic ulcerative syndrome-affected fish intramuscularly injected with *Aphanomyces invadans* NJM9701 zoospores. (a) Presence of Langhans type multinucleated giant cells (circles) in Goldfish (6 pi), H & E, 400×. (b) A number of granulomata (arrow) surrounded by fibroblast layers in climbing perch (10 pi), H & E, 400×. (c) Encapsulation fungi by Foreign body type giant cells (circle) and a granulomas (arrow) surrounding by thick fibroblast layers (4 pi), H & E, 400×. (d) Non-capsulated hyphae (arrow) in necrotic areas in Koi carp (18 dpi), P.A.S, 200×.

In snakehead, there was some hemorrhage in the epidermis and hyperemic blood vessels with fibroblast activity and myophagia at 24 h after injection. At day 2 of p.i., accumulations of mononuclear inflammatory cells in the dermis, separation of loose connective tissue of the dermis, and rupture of capillaries leading to hemorrhage were evident. The prominent feature as observed in infected fish was the degeneration of muscle tissues, blood vessels, and the presence of epithelioid cells. By the 4^th^ day, macrophages were present in a circular pattern of epithelioid cells circumscribing the lesion area and around the fungal hyphae, with some variance dependent upon fish species. At day 6-8 p.i., the regeneration of muscle fibers was active; the presence of fibroblast and granulation tissue were conspicuous in this period landscaped the entire defective area. From day 10-14 p.i., well developed small and large granulomata were dominant, composed of a few layers of epithelioid cells surrounded by variable thicknesses of fibrous encapsulations with a central area of the necrotic material. Generally, in other fish species except Nile tilapia, the histopathological pattern was similar, however with variation in granulomatous reaction, presence or lack of giant cells, and time of mortality. These were considered to reflect susceptibility (Table-1). In gourami (Figure-4b), koi carp (Figure-4c), and broadhead catfish (Figure-4d), the granulomas formation was weak and the oomycete fungi hyphae were observed as being distributed in most areas of the impairment with no granuloma. Whereas, in snakehead (Figure-4a), goldfish, and climbing perch (Figure-5b), the granulation process was well in progress, and epithelioid cells were accompanied by dense fibroblasts layers. At day 21 p.i., granulomata of varying sizes, mostly small sizes, were fused together in different areas, forming larger granulomata surrounded by layers of fibrous tissue. Degenerated fungal hyphae were observed in some granulomata. However, few free fungi hyphae were evident in the infected areas which were confirmed in PAS staining (Figure-5d). Formation of multinucleated giant cells was clearly evident in the infected area in catfish (Figure-4d), goldfish (Figure-5a), and Nile tilapia (Figure-5c), but not in other species.

**Table-1:** Characteristic histopathological findings compared among seven fish species infected with *Aphanomyces invadans*.

Histopathology findings fish species	Snakehead	Snakeskin gourami	Koi carp	Broadhead catfish	Goldfish	Climbing perch	Nile tilapia
Cellular infiltration	++	+	+	+	++	++	+++
Granulomas	Developed	Immature	Immature	Immature	Developed	Developed	Well developed
Giant cells	-	-	-	++	++	-	+++
Free hyphae	+	++	+++	++	+	+	-
Wound healing	+	-	-	-	+	+	+++

Note: Severity code: -=Absent, +=Mild, ++=Moderate, +++=Severe

In Nile tilapia, fully mature granulomata of varying sizes together with multinucleated giant cells were evident and filled the whole affected area in very early stages of infection. Most granulomata in varying areas fused together forming large granuloma (fusion) surrounded with dense fibrous layers and melanin pigments, and no free fungi hyphae were detected in infected tissues. At day 35 p.i., the tilapia’s lesion was healed, and regardless of the presence of granulomata, the microarchitecture of the tissue was almost identical to the normal tissue sample.

First mortality occurred in gourami and koi carp at day 7 p.i., which all fish died at day 14 p.i., and 18 p.i., respectively, showing lethargy and anorexia before death. Afterward, mortality was observed in catfish at day 8 p.i., in goldfish at day 10 p.i., in snakehead at 14 p.i., and all moribund fish were dead at day 21 p.i. The first climbing perch died at 15 p.i., and all the rest were found dead at day 28 p.i while Nile tilapia and fish of the control group did not show any mortality.

### Statistical analysis

The results of Chi-square test showed that there were significant clinical and pathological differences between control and experimental groups for all seven species of the examined fish (Table-2). The result of Kruskal–Wallis test showed that there was a significant difference in susceptibility between species, (*c*^2^=145.11, p<0.01). Examined fish including snakeskin gourami, koi carp, and broadhead catfish showed significantly higher than snakehead while lower significant were recorded in goldfish and climbing perch.

**Table-2:** Skin lesion score and significance of disease in *A. invadans* experimentally challenged fish.

Species/treatment	Skin lesion score					Mortality	χ^2^	p value

	0	1	χ^2^	3	4			
Snakehead								
Exp (%)	0/44 (0)	0/44 (0)	0/44 (0)	24/44 (54.5)	20/44 (45.5)	44/44 (100)	201.59	<0.001
Cntl	0/22	0/22	0/22	0/22	0/22	0/22
Snakeskin gourami								
Exp (%)	0/44 (0)	0/44 (0)	0/44 (0)	21/44 (47.7)	23/44 (52.3)	44/44 (100)	211.58	<0.001
Cntl	0/22	0/22	0/22	0/22	0/22	0/22
Koi carp								
Exp (%)	0/44 (0)	0/44 (0)	0/44 (0)	23/44 (52.3)	21/44 (47.7)	44/44 (100)	204.92	<0.001
Cntl	0/22	0/22	0/22	0/22	0/22	0/22		
Broadhead catfish								
Exp (%)	0/44 (0)	0/44 (0)	0/44 (0)	22/44 (50)	22/44 (50)	44/44 (100)	211.58	<0.001
Cntl	0/22	0/22	0/22	0/22	0/22	0/22		
Goldfish								
Exp (%)	0/44 (0)	0/44 (0)	6/44 (13.6)	22/44 (50)	16/44 (36.4)	44/44 (100)	176.95	<0.001
Cntl	0/22	0/22	0/22	0/22	0/22	0/22		
Climbing perch								
Exp (%)	0/44 (0)	0/44 (0)	9/44 (25.5)	20/44 (45.5)	15/44 (34.1)	44/44 (100)	167.97	<0.001
Cntl	0/22	0/22	0/22	0/22	0/22	0/22
Nile tilapia								
Exp (%)	39/44 (88.6)	5/44 (11.4)	0/44 (0)	0/44 (0)	0/44 (0)	0/44 (0)	22.5	<0.001
Cntl	0/22	0/22	0/22	0/22	0/22	0/22		

Cntl=Control, Exp=Experimental, Note: Figures in parentheses indicate percentage of each score and mortality, *A. invadans=Aphanomyces invadans*

## Discussion

In the current study, dermal ulceration and granulomatous reaction were successfully induced in the experimentally infected fish via i.m. injection of *A. invadans* live zoospores, the degree of which was similar to that would be expected due to natural EUS infection. It should be noted that the infection model used in the current study was not influenced by any biological or physiological factors, and the environmental factors were kept constant. The experimental fish received very similar doses of zoospores. Therefore, the fish’s immune response and anatomical factors were the main factors influencing the development of the disease. Although, the responses of the healthy fish under laboratory conditions may not be identical to that of fish infected naturally, it is nonetheless an important component of understanding the basic responses as evident in susceptible fish. The injected zoospores of *A. invadans* were able to grow in the muscles of experimentally infected fish and germinated into hyphae and proliferated extensively, causing necrotizing granulomatous dermatitis with the inflammatory response within a very short period after injection. However, of more than 100 fish species that have been reported to be affected by EUS, only a few of them had been validated by demonstrating the presence of mycotic granulomas in histological sections or by isolation of the pathogenic oomycete fungus, *A. invadans* from the tissues underlying the ulcers [2]. Experimental infection, as executed in USA with *A. invadans* which were carried out in four species of estuarine fish, led to similar lesions, and severe pathologies in Atlantic menhaden, *Brevoortia tyrannus*, and Killifish, *Fundulus heteroclitus* were observed [8]. These historical results supported the results of the current study which proved that some species of fish were more sensitive than others, and fish having soft epidermal skin layers such as gourami, ornamental carps, and catfish were more vulnerable to infection by *A. invadans*. Johnson *et al*. [8] stated that Atlantic menhaden have an oily flesh, a thin and fragile epidermal layer, and readily lose their deciduous scales that may facilitate the attraction of and infection by zoospores of *A. invadans*. Menhaden tissue has also been reported to support the increased growth of hyphae of *A. invadans* as compared with agar [9]. Thus, the predilection of *A. invadans* for Atlantic menhaden may also reflect the susceptibility of the host to stress, which facilitates entry, and by nature of the oily flesh, which serves as a significant supportive nutritional source for the oomycete [8]. Pradhan *et al*. [10] investigated the susceptibility of three species of Indian major carp (*catla, rohu*, and *mrigal*) and common carp to EUS and found that within a period of 12 days, there was a 100% mortality with severe gross lesion development and mycotic granulomatous lesions in Indian major carp, whereas, in common carp, neither mortality nor gross visible lesions were observed. Their findings, as well as histopathological examinations by Baruah *et al*. [11], inferred that advanced fingerlings of Indian major carp are highly susceptible to EUS. Oidtmann *et al*. [12] had conducted an experimental infection utilizing similar *A. invadans* strain NJM9701 and demonstrated that European catfish and *Silurus glanis* produced typical EUS ulcerative skin lesions. Three-spot gourami and *Trichogaster trichopterus* were used in their study as a positive control and demonstrated to be more vulnerable to *A. invadans* infection as compared to European catfish. Notwithstanding injected gouramies indicated the changed swimming behavior (forward and backward teethering movements) at day 6 p.i., however, in the current study similar swimming behavior were seen in gouramies at day 7 p.i. In the current study, observations of the inflammatory changes in fish after i.m. inoculation of *A. invadans* indicated the occurrence of the characteristic features of an acute to chronic inflammation. The main features of the chronic inflammation were cellular infiltration, hemorrhage, fibrosis, and vascularization in the lesion area at the early stages of the infection caused by the passage of the needle inserting the inoculums, and the zoospore activity which transmuted to complete degeneration of the muscle fibers. The main components of the inflammatory response were the inflammatory cells comprising mononucleated cells, giant cells, and fibroblasts. In this study, because of the presence of the pathogen in the tissue, inflammatory cells began to infiltrate into the lesion area resulting in the initiation of myophagia at 24 h after inoculation. Macrophages were observed in the lesion area from the early stages while their number and activity increased, but only the Nile tilapia succeeded to initiate the healing process, whereas, in the other examined fish, the number of inflammatory cells decreased at the final stages of sampling. The long-term activity and increasing number of macrophages could be an immunological reaction related to macrophages responsibility for the development of fish immunity [13]. However, such fish defense mechanisms deployed against the invading fungus were not effective enough to limit growth of the fungus inside the tissue of fish examined in this study except in respect to Nile tilapia which was known to be resistant to the EUS. In the current study, the presence of multinucleated giant cells with engulfed hyphae were observed in the lesion area in broadhead catfish, goldfish, and Nile tilapia at day 2, 6, and 4 p.i., respectively. Furthermore giant cells with no engulfed fungus were observed either in the lesion area or in the granulomata. The number and active giant cells significantly increased in Nile tilapia by the time, leading to encapsulation of fungi manifested in the infected area, however, this reaction in broadhead catfish and goldfish could not prevent fungal growth in the lesion. Other studies also reported the presence of giant cells in European catfish, *Silurus glanis* [12], goldfish, and other fish species such as Mirror carp [13], Ayu and Common carp [14], Atlantic menhaden, and striped killifish [6], but it was not reported in Snakehead as experimentally infected with *A. invadans* [15], dwarf gourami infected by *Aphanomyces* sp. [16], and in gray mullet or any other fish species. Thompson *et* *al*. [17] suggested that giant cell formation did not seem to be an indicator of resistance to EUS, since some of the highly susceptible species (Ayu, catfish, goldfish, and puntius) were capable of forming such, while, the actual impact of this type of response in respect to fungal infection is still unclear.

Mycotic granulomatous reaction which is an important defense mechanism of fish against EUS were observed in this study in all the experimentally infected fish, resulting in varying size and thickness indicated by epithelioid cells surrounding the fungal hyphae at days 6-8 p.i. Previous studies established that fibrous proliferation and intense granulomatous reactions in response to *A. invadans* may be an important factor inhibiting fungal growth. The results of this study demonstrated that granulomatous reaction could prevent and halt fungal growth only in resistant fish such as Nile tilapia, but in susceptible fish, for example snakehead, gouramies, koi carp, catfish, goldfish, and climbing perch, the rate of fungal growth was more rapid than granulomatous reaction and free fungal hyphae were detected in the infected area even at the time of mortality. In the current study, mortality in EUS susceptible fish was observed from day 7-14 p.i., however, thereafter life was prolonged as relative to a maximum of day 28 p.i. It was suggested that this delay in the time of mortality was due to some degree of resistance offered by the macrophages in the initial stages of infection [18]. During this period, the host may be attempting to prevent the invasive spread of the hyphae in tissue, however, in susceptible fish, the hyphae were able to invade neighboring tissues, proliferate [17] and cause extensive myonecrosis due to the release of proteolysins in large areas of myotome resulting in morbidity or death.

Although in Nile tilapia, the granulomatous reaction was well developed with a high level of solidification from a very early stage of disease (day 2 p.i.) leading to encapsulation of all fungi in infection area. From the findings of the current study, it could be suggested that cellular response of fish to mycotic infection and granulomatous reaction varied in different fish species which could not be an indicator of susceptibility or resistance to the EUS, however, the rate and the level of maturity found to be higher in resistant fish. These findings were similar to Wada *et al*. [14] who observed that EUS resistant common carp, *Cyprinus carpio*, developed granuloma far more rapidly than susceptible Ayu, *Plecoglossus altivelis*, without apparent gross manifestation. In contrast, Oidtmann *et al*. [12] did not observe any mycotic granulomas in artificially injected European catfish, *Silurus glanis*, with *A. invadans* isolate NJM 9701, which was the same fungal strain used in the current study, although the formation of multinucleated giant cells and granulomatous reaction in gouramies were recorded in their study. The present study and Phadee *et al*. [19] have succeeded in inducing *A. invadans* infection in goldfish, although Oidtmann *et al*. [12] failed to infect goldfish via intramuscular injection of *A. invadans* (NJM9701) zoospores. These results may suggest differences in pathogenicity of *A. invadans* strains on different host species, or the presence of differences in strain susceptibility among goldfish.

## Conclusion

From the findings of the current study, it was concluded that, compared to the EUS-susceptible snakehead and EUS-resistant tilapia, tropical snakeskin gourami, koi carp, and broadhead catfish were demonstrated to be highly susceptible, whilst goldfish and climbing perch were found to be moderately susceptible to the EUS infection. Histopathology examination was demonstrated to be practical and appropriate for detection of EUS infection within the 1^st^ day of the disease. Early detection and the application of suitable prophylactic methods can therefore circumvent early transmission, which if not caught and managed can lead to a serious outbreak of the disease and also the introduction of the pathogen into unaffected countries.

## Authors’ Contributions

SFA designed and carried out the experiment and drafted the final manuscript. HMD and IS analyzed the histological results. MA carried out the statistical analysis. SS was responsible for final editing and English proofreading of the manuscript draft. All authors read and approved the final manuscript.
